# Long-Distance Movement of mRNAs in Plants

**DOI:** 10.3390/plants9060731

**Published:** 2020-06-10

**Authors:** Chao Xia, Cankui Zhang

**Affiliations:** 1 Maize Research Institute, Sichuan Agricultural University, Chengdu 611130, China; chaoxia@sicau.edu.cn; 2 Key Laboratory of Biology and Genetic Improvement of Maize in Southwest Region, Ministry of Agriculture, Chengdu 611130, China; 3 Department of Agronomy, Purdue University, West Lafayette, IN 47907, USA; 4 Purdue Center for Plant Biology, Purdue University, West Lafayette, IN 47907, USA

**Keywords:** phloem, long distance, systemic, mobile mRNA, heterograft

## Abstract

Long-distance transport of information molecules in the vascular tissues could play an important role in regulating plant growth and enabling plants to cope with adverse environments. Various molecules, including hormones, proteins, small peptides and small RNAs have been detected in the vascular system and proved to have systemic signaling functions. Sporadic studies have shown that a number of mRNAs produced in the mature leaves leave their origin cells and move to distal tissues to exert important physiological functions. In the last 3–5 years, multiple heterograft systems have been developed to demonstrate that a large quantity of mRNAs are mobile in plants. Further comparison of the mobile mRNAs identified from these systems showed that the identities of these mRNAs are very diverse. Although species-specific mRNAs may regulate the unique physiological characteristic of the plant, mRNAs with conserved functions across multiple species are worth more effort in identifying universal physiological mechanisms existing in the plant kingdom.

## 1. Introduction

Higher plants have evolved a communication system that enables the coordination of developmental cues and environmental inputs [1,2]. The local communication is achieved between different cellular compartments inside a cell or adjacent cells by symplasmic transmit through plasmodesmata, while inter-organ communication is realized by long-distance signaling that takes place in the vasculature [3,4]. Phloem, one of the major components in the vasculature system, has long been recognized as a tissue that transports carbohydrates and amino acids. In recent years, it has been found that phloem harbors a diverse population of components, e.g., mRNAs, small RNAs, proteins, small peptides and hormones [5].

While scientists have had a relatively comprehensive understanding of the biological functions of many phloem located, long-distance mobile molecules, little is known on the importance and breadth of mRNAs in participating in the systemic signaling and regulation of plant physiology. mRNAs have been traditionally viewed as local intermediate components between the genomic DNAs and the proteins in a cell. However, this conventional view of mRNAs has been challenged because a handful of studies have shown that mRNAs produced in mature leaves can leave their original cells and move to distal tissues via the phloem to exert plant physiology functions [3,6]. For example, the long-distance movement of *AtIAA18*, *AtIAA28* and *AtTCTP1* mRNA is indispensable for the root development in Arabidopsis [7,8,9]; although FT (Flowering Locus T) protein is demonstrated to be the systemic florigenic signal, long-distance movement of *AtFT* mRNA from mature leaf to shoot apex has also been suggested to be involved in flowering initiation in Arabidopsis [10,11]; similarly, the mRNAs of a few *FT* homologs, *NsSP3D*, *NsSP5G*, and *NsSP11A,* are required in the flowering induction in *Nicotiana Sylvestris* [12]; in potatoes, a number of mRNAs, i.e., *StBEL5*, *StBEL11, StBEL29* and *StPOTH1*, have been found to be able to move from mature leaves to stolon for tuberization initiation and storage root development [3,13,14,15]; in tomatoes, the long-distance movement of *PFP-LeT6* and *SlPS* mRNAs is essential for tomato leaf development [16] and resistance to the necrotrophic fungus *Botrytis cinereal* [17], respectively. These pioneer studies conducted in the last two decades demonstrated the importance of long-distance movement of mRNAs in physiological regulation; however, how common this phenomenon is for other mRNAs remains an intriguing question until the recent development of a few systems that enabled the discoveries of large-scale mobile mRNAs in plants.

## 2. Identification of Large-Scale Movement of mRNAs Using Heterograft Methods

Three major sampling methods, i.e., stylectomy, EDTA facilitated, and cucurbit bleeding, can be used to study mobile mRNAs in the phloem. However, the lengthy sampling procedure associated with the stylectomy method, the possibility of contaminations derived from the damage of EDTA to plant tissues, and the non-phloem origin of most of the cucurbit “phloem” sap make these methods less ideal in dissecting phloem mobile molecules [18,19]. In the last 3–5 years, important breakthroughs have been made in the area of mRNA movement using other alternative approaches. One of the most prominent discoveries was derived from a system in which parasitic plants and their hosts were used. It was found that a total of 9518 mRNAs (45% of the whole genome) from the host Arabidopsis moved into the parasitic *Cuscuta*; meanwhile, 8665 mRNAs from the parasitic *Cuscuta* moved into the Arabidopsis [20]. On the contrary, when parasitic *Cuscuta* was attached to the tomato host, only 347 tomato mRNAs moved into *Cuscuta*, and 288 *Cuscuta* mRNAs moved into the tomato. In addition to the host parasitic system, a few heterograft systems, in which one species or genotype is grafted onto the other species or genotype, were used to demonstrate the existence of the large scale, vasculature-mediated movement of mRNAs between different organs, as shown in Table 1. For example, Thieme et al. (2015) determined that 2006 mobile mRNAs moved from the shoot to the root or vice versa, in which two ecotypes of Arabidopsis were used as either scion or rootstock [21]; Notaguchi et al. (2015) identified 138 Arabidopsis mobile transcripts in the scion in a *Nicotiana benthamiana*/Arabidopsis heterograft system [22]; Yang et al. (2015) reported that 3333 mRNAs were transmittable within a heterograft system consisting of two grape varieties [23]; Zhang et al. (2016) revealed that 3546 mRNAs moved from the cucumber mature leaves into watermelon sink tissues [24]; Wang et al. (2020) identified 2386 mobile mRNAs in a watermelon/bottle gourd heterograft system [25]; we (2018) detected 1063 shoot-to-root mobile mRNAs in which the *N. benthamiana* plant was used as scion and the tomato was used as rootstock [26]. Comparisons of the mobile mRNAs from these systems showed that the identities of the mRNAs are highly species specific [26,27]. This indicates that a better understanding of the basic physiological processes, e.g., cellular origin, destination, related to mRNAs is needed before large-scale functional characterization of these mRNAs at the molecular level are pursued.

### 2.1. Cell Origin of Mobile mRNAs

The aforementioned studies have ambiguously demonstrated that certain mRNAs produced in the leaf can move to other distal organs via phloem. Due to the complicated cell ultrastructure and diverse cell types in leaf, it is legitimate to assume that not all leaf cells contribute equally in the biosynthesis and transport of mRNAs. Evidence from our study [26] and Thieme et al. [21] demonstrated that the mobile mRNAs detected in the heterografts were over represented in previously identified phloem mRNAs derived from other methods. Via a computational analysis, Calderwood et al. [28] also concluded that mRNAs located in the phloem companion cells had a higher possibility to move.

**Figure 1:**
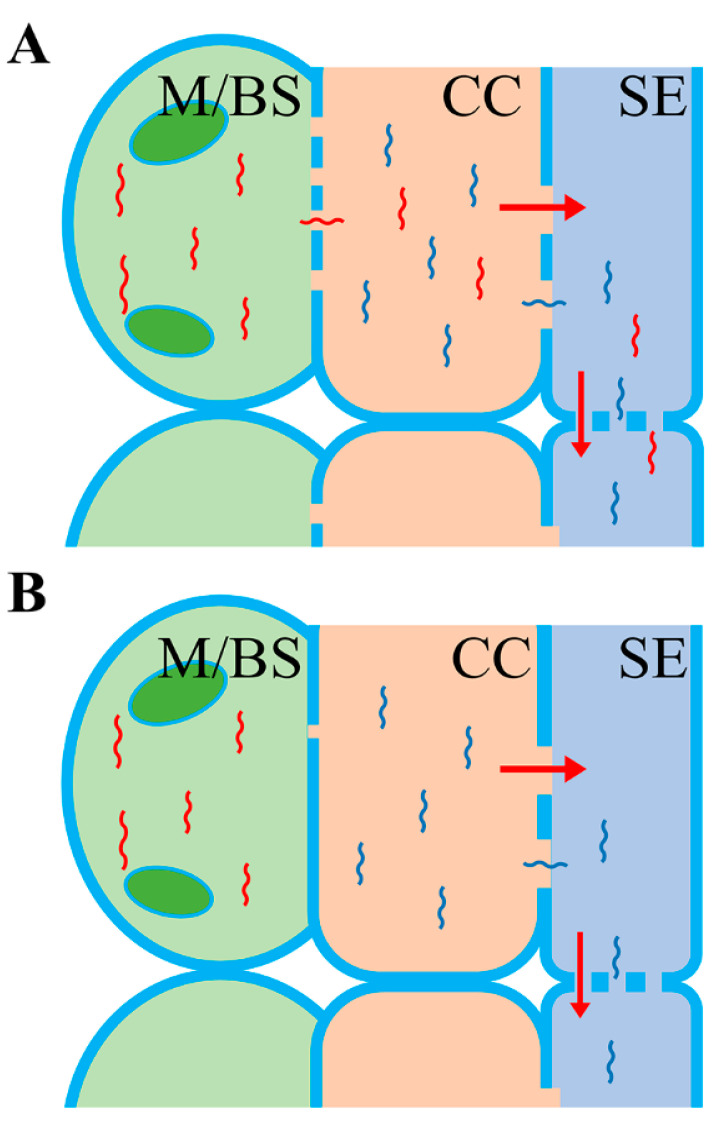
Postulated model related to the origin of mobile mRNAs in woody and herbaceous plants. (**A**) The abundant plasmodesmata between the M/BS and CC in woody species allow the movement of mRNAs transcribed in the M/BS cells to the CC–SE. mRNAs transcribed in the CC also have the potential to move to the SE for long-distance transport. (**B**) The very rare and narrow plasmodesmata between the M/BS and CC in the herbaceous species restrict the movement of mRNAs transcribed in the M/BS cells to the CC. Only mRNAs transcribed in the CC have the potential to move to the SE for long-distance transport. M/BS: mesophyll cells or bundle sheath cells; CC: companion cell; SE: sieve element.

Another interesting discovery was from Yang et al. [23] who found that among the most abundant 33 leaf mRNAs identified from the grapevine grafting system, approximately half (17) of them moved over a long distance. This finding implied that the abundance of mRNAs in leaves has a high correlation with movement. However, in our *N. benthamiana*/tomato heterografting system, we found that the abundance of mRNAs in leaves had no correlation with the mobility [26]. In our system, none of the 100 most abundant mRNAs in the leaf moved to the rootstock. What is the explanation of the big difference between our discovery and the one from Yang et al.? We believe that the anatomical structural difference of leaf cells between herbaceous (our system) and woody species (system in the Yang study) may play a role in the generation of this distinction, as shown in Figure 1.

In herbaceous species, there are very limited plasmodesmata connections between the phloem cells, which are involved in long-distance transport and signaling, and the other surrounding leaf cells, such as bundle sheath and mesophyll cells [29]. On the contrary, in woody species, the phloem and its surrounding leaf cells are connected with a high abundance of plasmodesmata [29]. At present, it has been widely accepted that such structural characteristics in herbaceous and woody plants are associated with specific transport mechanisms for small molecules, such as carbohydrates [29,30,31]. In herbaceous species, sugars produced in mesophyll cells during photosynthesis cannot directly move into the phloem through plasmodesmata, due to the low abundance of plasmodesmata. Sugars have to be exported to the apoplast of the phloem and then taken up by sugar transporters localized on the plasma membrane of phloem companion cells (CC). In woody plants, this apoplast step is not needed because sugars produced in mesophyll cells can be directly transported to the phloem via the abundant plasmodesmata [26,29]. The discrepancy between our observation and that from Yang et al. indicated that the aforementioned theory related to sugar movement may also apply to mRNAs. In most plants, phloem cells (including companion cells and sieve elements (SE)) account for only 1 to 3% of all the leaf cells [29]. Therefore, highly expressed mRNA populations identified by RNA-seq or quantitative reverse PCR in leaves are more likely to be expressed in all leaf cells. In woody plants (such as grapevine), these high-abundance mRNAs, regardless of their cell types, are likely to move because of the rich plasmodesmata among all the cells in leaf, as shown in Figure 1A. Therefore, a strong correlation between the abundance and movement is more obvious. However, the scenario may be completely different in a system involved in herbaceous species (such as our *N. benthamiana*/tomato heterograft system). The high-abundance mRNAs, unless located in phloem, are not prone to move due to the low density of plasmodesmata between phloem and the surrounding cells, shown in Figure 1B. Therefore, a positive correlation between the mRNA abundance and mobility is less likely to appear. In agreement with this hypothesis, a study suggested that the long-distance movement of *AtGAI* mRNA was compromised when it was ectopically expressed outside of the phloem in Arabidopsis [32].

### 2.2. Destination of Mobile mRNAs in the Root

When leaf-produced mRNAs arrive in root via phloem, they or the translated proteins derived from these mRNAs need to be unloaded from the sieve tube to other cells to exert physiological functions. The root system in plants is complex and consists of multiple types of cells, e.g., phloem, xylem, cortex, endodermis, epidermis and pericycle. [33]. Similar to the limited exploration on the cellular origin of the mobile transcripts, very few efforts have been focused on the identification of the recipient cells in roots. A previous study on *StBEL5* mRNA indicated that the root has a mechanism to distinguish the shoot-born mRNAs because the mRNA transcript is only enriched in stolon. This tissue-specific distribution of the *StBEL5* mRNA in underground tissue is important for the initiation of tuberization and tuber development [3,34]. The enrichment mechanism is not clear but two possibilities exist. *StBEL5* mRNAs could be specifically unloaded to stolon as suggested by Banerjee et al. [3], or the transcripts were unloaded to all root cells but a cell distinctive mRNA degradation mechanism is involved that leads to the low abundance of mRNAs in cells with high mRNA turnover activities.

Recently, Ross-Elliott et al. [35] established a mathematical model and suggested that phloem unloads solutes through the symplasmic plasmodesmata connection into the adjacent phloem pore pericycle (PPP), by a combination of mass flow and diffusion in Arabidopsis roots. While small solutes such as sugars are exported without restriction, macromolecules (e.g., proteins) are restricted in the PPP due to the size-dependent filtration [35]. Experimental evidence supporting the mathematical model came from a study conducted by Yang et al. [8]. It was found that *YFP-TCTP* mRNA can be translated into its corresponding YFP-TCTP protein after they arrive in the root. However, YFP-TCTP proteins were mainly observed in PPP but totally absent in other root cells such as xylem pore pericycle (XPP) [8]. It is important to realize that the specific localization of YFP-TCTP protein in PPP cells does not restrict its physiological effect to be solely in these recipient cells because the movement of *AtTCTP* from the shoot to the root promoted the initiation of lateral root primordia, a tissue different from PPP [8,9]. A previous study has shown that LR primordia of Arabidopsis arises in the XPP [36], a cell that does not directly receive the TCTP protein [8]. The underlined mechanism is not clear but one possibility is that the arrival of the TCTP proteins in PPP cells stimulates a cascade of molecular processes in which certain downstream components further move from PPP to XPP to initiate the primordia formation.

### 2.3. Factors Conferring Mobility

All the heterografting systems demonstrated that only a portion of the transcribed mRNAs in the leaf can move to the root. This indicates that certain factors associated with these mRNAs are related to their mobility. As discussed above, it was suggested that mRNAs with high abundance in the phloem companion cells are more prone to move [28]. If abundance in the companion cells is the only factor conferring mobility, then transcripts, including those that are not mobile, should move if their abundances are increased in the companion cells. Our research showed that this is not the case. *AtAMT1*;2 and *AtCHL1* are two transcripts that were identified to be immobile in all the published heterograft systems. When these two mRNAs were individually overexpressed in the companion cells driven by a companion cell-specific promoter in potato, the lack of detection of these mRNAs in the root from the heterografts in which the transgenic plants were used as scion and wild type as rootstock, suggested that increasing abundance solely was not enough to promote the mobility of these two transcripts. It should be noted that our result does not negate the importance of mRNA abundance in companion cell in conferring mobility. Instead, it was suggested that other factors were also involved in regulating mRNA movement [37].

In addition to abundance, another factor that may participate in the regulation of mobility is the plasmodesmata between the companion cells and the sieve element. The size exclusion limit (SEL) of these plasmodesmata in the collection phloem of leaf is reported to be ~67 kDa in Arabidopsis [38]. Small molecules, such as sugars and amino acids, can move freely through these plasmodesmata but the movement of macromolecules larger than the SEL may be restricted [38,39]. For example, when *GFP* is overexpressed in the companion cells, the protein of GFP can enter the SE for long-distance movement, while the mRNA of *GFP* cannot [39]. Although the lack of endogenous RNA-binding protein for *GFP* transcript may be one reason related to this observation, the large size of *GFP* mRNAs (230 kDa), which is beyond the SEL of the plasmodesmata, could restrict the *GFP* mRNAs in the companion cells.

Multiple studies have indicated the importance of specific sequence motifs in mRNAs in conferring mobility. Similar to the lack of mobility of GFP mRNAs, *GUS*, with a molecular mass at 652kDa, was not mobile when it was overexpressed by the 35S promoter. However, the mobility of *GUS* can be achieved if a *TLS* (tRNA-like sequence) motif is fused with *GUS* [40]. An analysis of the mobile mRNAs from most of the published systems showed that only 10–15% of these transcripts harbor TLS. This indicates that other motifs may also be involved in transcript mobility. Banerjee et al. [34] demonstrated that both 5’ and 3’ untranslated regions of the mRNAs of *StBEL5* were involved in mediating long-distance transport from shoot to stolon. A cis-acting element required for RNA mobility was mapped to the coding region of *AtFT* mRNAs [41]. The sequence of *AtGAI* at coding and 3’ untranslated regions constitutes the motifs necessary for RNA movement [32]. In addition, Yang et al. showed that the 5-methylcytosine (m5C) modification of mobile mRNAs plays a crucial role in facilitating their transport [8]. 

It has been suggested that the movement of RNAs is facilitated by RNA-binding proteins (RBPs) and specific motifs located in different mRNAs can be recognized by certain RBPs [13]. For example, the 3’ UTR of *StBEL5* harbors a poly-pyrimidine sequence element that specifically interacts with polypyrimidine tract-binding proteins (PTBs) in potatoes [42]. It is known that the RNA–RBP complex not only facilitates movement, but it also protects the RNA from degradation. In our study, we found that some of the leaf-born mRNAs had a higher abundance in roots, but most of them were degraded during their shoot-to-root movement [26]. It remains interesting to explore whether the degradation of the mobile mRNAs was due to the lack of RBPs to them [43,44]. Other potential routes of mRNA transport to enter the phloem, e.g., via vesicles, have also been proposed [45].

### 2.4. Physiological Functions of the Mobile mRNAs

Various studies have demonstrated that the long-distance movement of mRNAs may be associated with important physiological processes, such as root development, flowering, tuberization and leaf development. Notaguchi et al. [7] found that *AtIAA18*, *AtIAA28* transcripts were synthesized in the vascular of mature leaves and their movement to the root regulated lateral root development. Yang et al. [8] determined that the 5-methylcytosine (m5C) modification of *AtTCTP1* mRNA was required for its transport and the movement of m5C-modified *TCTP1* mRNA was essential for root growth. Branco and Masle [9] also revealed that the long-distance transported *AtTCTP1* mRNA specifically stimulates the emergence of the lateral root along the primary root pericycle, while the root elongation is partially controlled by the local constitutive *TCTP1* expression. The *AtFT* mRNA functions as a systemic floral signaling to promote vegetative-to-reproductive transition in Arabidopsis [10]. The FT belongs to phosphatidylethanolamine-binding domain protein (PEBP) family. Further study suggested that a number of members in the PEPB family, such as *NsCET1, NsSP9D, NsSP3D*, *NsSP5G*, *NsSP11A*, and *NsSP2G*, are phloem mobile [12]. Potato tuberization is also controlled by mobile mRNAs. The potato *StBEL5* mRNA was demonstrated to be a long-distance signal that is expressed in the phloem cells of leaves and transmitted into roots and stolons to initiate tuberization [3,14]. The overexpression of *StBEL5* mRNA using a leaf-specific promoter helped overcome the inhibitory effects of long days on tuber formation and enhanced the tuber yield [3]. Phloem-mobile *StPOTH1* mRNA functions synergistically with *StBEL5,* while *StBEL11* and *StBEL29* functions antagonistically to *StBEL5* [13,14,15]. The long-distance movement of mRNAs also controls leaf development. Kim et al. [16] reported that the chimeric *PFP-LeT6* fusion mRNA was transported from the mutant rootstock to the heterografted wild-type scion and caused the leaf’s morphological changes in the scion. *AtGAI, CmGAIP* and *CmNACP* were found to be mobile and regulated the shoot apex development in Arabidopsis [46] and pumpkin [47]. A study has also shown that CmPP16 functions as an RNA-binding protein that carries various mRNA molecules from companion cells to the sieve element. The long-distance movement of tomato *SlPS* mRNAs in Arabidopsis was essential for resistance to the necrotrophic fungus [17].

To our knowledge, the aforementioned list of mRNAs were the major ones for which in-depth functional characterizations have been conducted. However, in recent years, hundreds of mRNAs have been identified to be mobile from the Arabidopsis, grapevine, cucumber and *Solanaceae* grafting systems, respectively [21,23,24,26]. It is legitimate to assume that mRNAs with conserved biological functions should exist in most, if not all, of these systems. However, we compared the mobile mRNAs and found only one “core” mRNA shared by all the systems [26]. This indicated that either most of the mobile mRNAs have no functions or they play species-specific functions in plant physiology. In addition to the *N. benthamiana*/tomato heterografting system, we also developed another system in which canola was the scion and *Arabidopsis* was the rootstock [26]. The extremely low number, i.e., twenty-three, and the high variation of mobile mRNAs identified from this system further indicated that more understanding is needed from the perspective of these mobile mRNAs as a population before in-depth molecular functional characterizations of individually selected mobile mRNA was pursued.

If some of the mobile mRNAs do not have specific physiological functions, why do plants generate and transport such a large population of mRNAs? Two mechanisms, i.e., selective and non-selective, have been proposed to be used by plants to transport their mRNAs [28,40,48]. For mRNAs with important physiological roles in the distal tissues, the generation and movement should be selective and tightly regulated. For example, a mRNA has to harbor a specific motif or be of a certain length; however, for mRNAs that are produced in the leaf, particularly companion cells, at high abundance but without essential features for long-distance movement, they may still enter the phloem sieve tube translocation stream. These mRNAs may be completely or partially degraded during their movement. To test this possibility, we designed a *N. benthamiana*/tomato heterograft system in which the stem of the tomato rootstock was 2.5 m long. We discovered that a total of 1,096 *N. benthamiana* mRNAs passed the graft joint, but 854 of them disappeared during their movement from shoot to root [26]. It is reasonable to assume that the movement of mRNAs undergoing degradation in the phloem is not selective and regulated; therefore, it is less likely that there are physiological functions associated with them. An alternative way to interpret this phenomenon is that plants use the degradation mechanism to remove excess cellular mRNAs. In addition, Melino et al. suggested that the turnover of RNAs and the catabolism of nucleotides may supplement the internal nitrogen pool and support the growth of the plant [49].

### 2.5. Methods to Identify Mobile mRNAs

Previous studies using EDTA-facilitated exudation or cucurbit exudation identified large numbers of mRNAs in the phloem translocation stream [50,51]. However, the authenticity of these identified mRNAs was often questioned [5,18,19,31]. The recent adoption of the multiple heterograft systems significantly improved the authenticity and increased the numbers of identified mobile mRNAs. Nonetheless, attention must be paid when using heterografts for mobile mRNA identification. Each of these steps, e.g., finding the two compatible species, sampling, the preparation of libraries and the next-generation sequencing, and data mining, could lead to false discoveries.

The two species used in the heterograft should be elaborately selected [52]. In general, these criteria should be satisfied: a) the two species should be phylogenetically close enough so they can be grafted; b) the genome sequences for the two species should be distant enough so the mRNAs can be unambiguously assigned to one species or another. For example, in the *Arabidopsis* Col/*Arabidopsis* Ped heterograft system, it was predicted that 72% of mobile mRNAs were not able to be identified due to the high genome-sequence similarities between the two ecotypes [21]. On the contrary, in the *N. benthamiana*/*Arabidopsis* system, the highly different genome sequences between the two species was an advantage in the identification of mobile mRNAs; however, the two species involved belong to different families. This may lead to strong physiological distortion and some of the identified mRNAs may be related to the non-native physiological alterations. Indeed, a number of the mobile mRNAs identified from this system are related to stress responses [22].

One of the essentials shared by all these heterografts is the adoption of the RNA-Seq for the exhausted identification of mRNAs transmitted between the two plants. Two major analytical methods were developed to facilitate the analysis. Thieme et al. [21] and Yang et al. [23] used SNPs in their systems in which different genotypes of the same species were used; while other investigators directly compared genome sequences for the identification because species with more distant relationships were used in their grafting systems [20,22,24,26]. Due to the close relationship and similar genome sequences of the two genotype/species in the heterografts, it is extremely important to apply strict informatics procedures so false discovery can be avoided. First, RNA-Seq libraries prepared from the source and the recipient tissues should be loaded into different lanes for sequencing. This will eliminate the possibility of crosstalk between samples even if various adapters are used to index samples during library construction. Second, a homograft in which both the scion and rootstock are the recipient species in the heterograft system should be established. This homograft is used as a control to eliminate false positives derived from the heterografts.

## 3. Future Perspective

Thousands of mRNAs have been identified to be mobile from the various heterograft systems. The functional characterization of individual mRNAs should be pursued in the near future. However, it is equally important to elucidate the general mechanisms related to the movement of these mobile mRNAs. For example, do herbaceous and woody plants differ in the origin of mobile mRNAs? To address this question, a mobile mRNA fragment, e.g., *GFP+TLS*, can be overexpressed either in the phloem or outside of the phloem in herbaceous and woody plants. The transgenic plants can be grafted on the non-transgenic rootstock and the abundance of *GFP* transcripts can be measured in the rootstock. A high abundance of the *GFP* will indicate the successful export of this transcript from the overexpressed cells. When sugars arrive in roots, they need to be unloaded from phloem to other root cells for metabolism. Do the shoot-to-root mobile mRNAs need to be unloaded, and if so, what are the recipient cells? In the heterografting system, usually ~30 m reads on the whole root tissue were sequenced for mobile mRNA discovery. The depth of single-cell sequencing can now reach as high as 15–30 m reads for each cell type and it can be used to locate cell-specific shoot born-mRNAs. A third question that is interesting to ask is the integrity of the mobile mRNAs. Our *N. benthamiana*/tomato heterograft system with a 2.5 m long stem let us discover that degradation occurs during the movement of mRNAs. However, partially degraded mRNAs could still be caught by RNA-Seq and assigned to be mobile. Therefore, it remains extremely important to study the integrity of a mRNA before any in-depth functional characterization is pursued. Other intriguing questions that remain to be answered include the identification of factors conferring the mobility and proteins that bind to the mobile mRNAs. The last, but not the least interesting and challenging question, is the differentiation of the effect between the mobile mRNAs and their corresponding proteins in the recipient organs.

## Figures and Tables

**Table 1:** Overview of the mobile mRNA identified in multiple heterografts.

Quantity of mRNAs	Scion	Rootstock	mRNA Origin Tissue	mRNA Recipient Tissue	Reference
1698	Arabidopsis	Arabidopsis	Shoot	Root	[21]
1032	Arabidopsis	Arabidopsis	Root	Shoot	[21]
138	*N. benthamiana*	Arabidopsis	Mature leaf/root	Stem	[22]
1963	Grapevine	Grapevine	Shoot	Root	[23]
2210	Grapevine	Grapevine	Root	Shoot	[23]
2682	Watermelon	Cucumber	Mature leaf	Developing leaf	[24]
471	Watermelon	Cucumber	Mature leaf	Shoot apex	[24]
1593	Cucumber	Watermelon	Mature leaf	Root	[24]
854	*N. benthamiana*	Tomato	Mature leaf	Root	[26]
283	*N. benthamiana*	Tomato	Mature leaf	Stem	[26]
1159	Watermelon	Bottle gourd	Shoot	Root	[25]
1,233	Watermelon	Bottle gourd	Root	Shoot	[25]
